# Graphene-Assisted Sensor for Rapid Detection of Antibiotic Resistance in *Escherichia coli*

**DOI:** 10.3389/fchem.2021.696906

**Published:** 2021-05-31

**Authors:** Chunlei Li, Feng Sun

**Affiliations:** ^1^ Department of Gastroenterology, Jiaozhou Central Hospital, Jiaozhou, China; ^2^ Department of Colorectal and Anal Surgery, The First Affiliated Hospital of Guangzhou University of Traditional Chinese Medicine, Guangzhou, China

**Keywords:** electrochemical sensors, drug-resistant strains, activity determination, antibiotics, electrode modification, catalytic reduction current

## Abstract

In recent years, antibiotic-resistant bacteria caused by antibiotic abuse in the medical industry have become a new environmental pollutant that endangers public health. Therefore, it is necessary to establish a detection method for evaluating drug-resistant bacteria. In this work, we used *Escherichia coli* as a target model and proposed a method to evaluate its drug resistance for three antibiotics. Graphene dispersion was used to co-mix with *E. coli* cells for the purpose of increasing the current signal. This electrochemical-based sensor allows the evaluation of the activity of *E. coli* on the electrode surface. When antibiotics were present, the electrocatalytic reduction signal was diminished because of the reduced activity of *E. coli*. Based on the difference in the electrochemical reduction signal, we can evaluate the antibiotic resistance of different *E. coli* strains.

## Introduction

Antibiotics are secondary metabolites that can interfere with cell growth and development (Simioni et al., 2017; Wang M. et al., 2019). They are mainly of microbial origin. The biochemist Fleming first discovered penicillin in 1929. Penicillin played an important role in World War II and was very effective in controlling bacterial infections (Alsaiari et al., 2021). However, the harm of antibiotics to the human body should not be underestimated. For example, furacilin enters the human body through food and may cause cancer with long-term consumption (Hu et al., 2010). Similarly, the commonly used sulfonamide antibiotic sulfadimethoxine has tumorigenic effects (Zhuang et al., 2019). According to the classification of chemical structure, antibiotics can be roughly divided into quinolone antibiotics, sulfonamide antibiotics, chloramphenicol antibiotics, aminoglycoside antibiotics, beta-lactam antibiotics and tetracycline antibiotics (Sharaha et al., 2017).

Large amounts of antibiotics are often used in the medical industry, and bacteria can develop resistance under the pressure of antibiotic selection. Antibiotic resistance genes (ARGs) are intrinsic to the development of drug resistance (Osman et al., 2021). Earlier studies have found that resistant bacteria are able to transfer the resistance genes they contain to other bacteria through animal excreta at the genetic level, eventually causing the large-scale presence of resistant bacteria (Hu et al., 2017).

Drug-resistant bacteria (ARB) are some bacteria that are originally sensitive and turn out to be resistant to drugs (Bengtson et al., 2017; Mulat et al., 2019). However, in low concentrations of antibiotics, some bacteria that were previously resistant tend to lose their resistance. This is because sensitive bacteria require fewer nutrients than resistant bacteria and have an advantage when competing with resistant bacteria, which inhibit the growth of resistant bacteria (Mishra et al., 2018; Sun et al., 2020). Therefore, reducing the abuse of antibiotics can reduce the risk of drug resistance. In general, long-term use of antibiotics tends to lead to the development of bacterial resistance (Gorlenko et al., 2020; Karimi-Maleh et al., 2020; Zhao et al., 2020). Bacterial resistance has become one of the top 10 global health threats, and its contamination is widespread and persistent. ARGs enter the human body through the food chain and cause an imbalance in the normal flora and increase the resistance of pathogenic and conditionally pathogenic bacteria in the body, posing a serious threat to the health of the body and disease control (Wang Y. et al., 2019; Karimi-Maleh et al., 2021a).

The traditional method for detection of bacterial resistance is the microbial inhibition method. Traditional microbial suppression methods are mostly based on bacterial isolation and culture methods, mainly for the detection of bacterial drug resistance phenotypes (Asghar et al., 2017; Zhou et al., 2017; Karimi-Maleh et al., 2021b, 2021c). The commonly used detection methods are mainly paper diffusion method and agar dilution method. The paper diffusion method is to apply a drug-sensitive tablet to M–H agar that has been inoculated with the bacteria to be tested, and then measure the inhibition circle after incubation. The size of the inhibition circle is closely related to the resistance of bacteria, and the strength of bacterial resistance to antibiotics is analyzed according to its size (Sedki et al., 2017). Polymerase chain reaction (PCR) is a molecular biology technique that allows rapid amplification of target genes. Compared with traditional microbial inhibition methods, this technique has the advantages of being less time-consuming and easier to perform, and it can also meet the requirements of simultaneous detection of large quantities of samples (Bagheri et al., 2019; Phung et al., 2020). However, the PCR technique also has some shortcomings, such as easy contamination. Even a very small amount of contamination can still cause false positives (Wu et al., 2020; Zhang X. et al., 2020; Zhi-bin et al., 2021). Moreover, this technique is limited by the design of primers. Quantitative real-time fluorescent PCR (qPCR) is based on normal PCR, where a fluorescent dye or probe is added to the PCR reaction system to reflect the amount of PCR product in real time by changes in fluorescence signal. During the qPCR process, the entire process is monitored in real time, allowing the quantification of the amount of starting template (Waseem et al., 2019). The method is more specific, but expensive and not suitable for analysis of a large number of samples. Therefore, it is necessary to develop a rapid way to evaluate bacterial resistance.

In recent years, there have been recent advances in the electrochemical ultrasensitive detection of bacteria (Farooq et al., 2020; Fu et al., 2020; Khan et al., 2020; Zhang L. et al., 2020). The principle of electrochemical methods for detecting bacterial drug sensitivity is that bacterial respiration relies mainly on electron transfer in the respiratory chain, and the coincidental introduction of redox probes intervenes in the bacterial respiratory chain. The electrochemical changes generated by the respiratory chain activity can be detected rapidly and reliably by electrochemical methods. Ertl et al. (2000, 2003) used potassium ferricyanide as a redox probe. *Escherichia coli* was mixed with a solution of potassium ferricyanide after 15 min of interaction with antibiotics, and the electrical signal was measured by the chronoelectric method. The results were in complete agreement with the conventional paper diffusion method. This method can provide a report in <25 min. However, the IC_50_ values measured by electrochemical method were 100 times higher than the results obtained by standard turbidity method, and the electrodes were found to adsorb antibiotics during the test. Chotinantakul et al. (2014) improved the test protocol based on Ertl et al. The antibiotics were removed by centrifugation of *E. coli* after interaction with bacteria and resuspended in a test solution containing potassium ferricyanide, which gave the results of the drug sensitivity test in 3–6 h.

However, the detection of bacterial resistance using conventional commercial electrodes has the disadvantage of insufficient sensitivity. Therefore, improving the performance of electrodes can be a good way to improve the accuracy of detection. In this work, we modified the conventional glassy carbon electrode (GCE) with surface graphene ink, which can greatly improve the sensing performance of the electrode (Baghayeri, 2017; Zhang M. et al., 2020; Mohanraj et al., 2020; Xu et al., 2020). The modified electrode can detect the electrochemical reduction behavior of *E. coli* more sensitively. Likewise, the differences in the altered electrochemical behavior were amplified due to the influence of different antibiotics after This technique could potentially be applied for the evaluation of resistance for *E. coli*.

## Materials and Methods

All electrochemical measurements were carried out using a CHI660E working station. A three-electrode system was applied for all measurements. Specifically, a glassy carbon electrode (GCE), a Pt foil and an Ag/AgCl electrode were used as working electrode, counter electrode and reference electrode, respectively. *Escherichia coli* J53 was purchased from Beijing Bio Bo Wei Biotechnology Co., Ltd. Ofloxacin, penicillin and cefepime was purchased from Sinopharm Chemical Reagent Co., Ltd. Graphene dispersion was purchased from Jiangsu XFNANO Materials Tech Co., Ltd. All other reagents used in this work were analytical grade and used without further purification. Phosphate buffer solution (PBS, 0.1 M) was prepared by mixed stock solutions of 0.1 M disodium hydrogen phosphate and sodium dihydrogen phosphate until reach to the desired pH.

*Escherichia coli* J53 was grown over night in a Luria Bertani (LB) medium (100 ml) at 37°C with shaking. The cells of *E. coli* were collected after centrifugation and washed by PBS. The colony forming units (CFU) were then counted. Then, the *E. coli* was diluted by graphene dispersion to reach a desired CFU by stirring.

Electrode surface modification was conducted by drop coating of desired concentration of graphene-*E. coli* dispersion on the GCE surface and kept in a humid chamber for 1 h before analysis. Then, the electrode was inserted into a PBS and conducted a voltammetric scan. The *E. coli* modified GCE was prepared using a similar method but with out the mixing of graphene dispersion.

For antibiotic resistance tests, 5 μL of ofloxacin, penicillin and cefepime solution was drop coated at graphene-*E. coli* modified GCE. Then, the electrode was kept in a humid chamber. The viability test was carried out at 1 h interval.

## Results and Discussion

**FIGURE 1:**
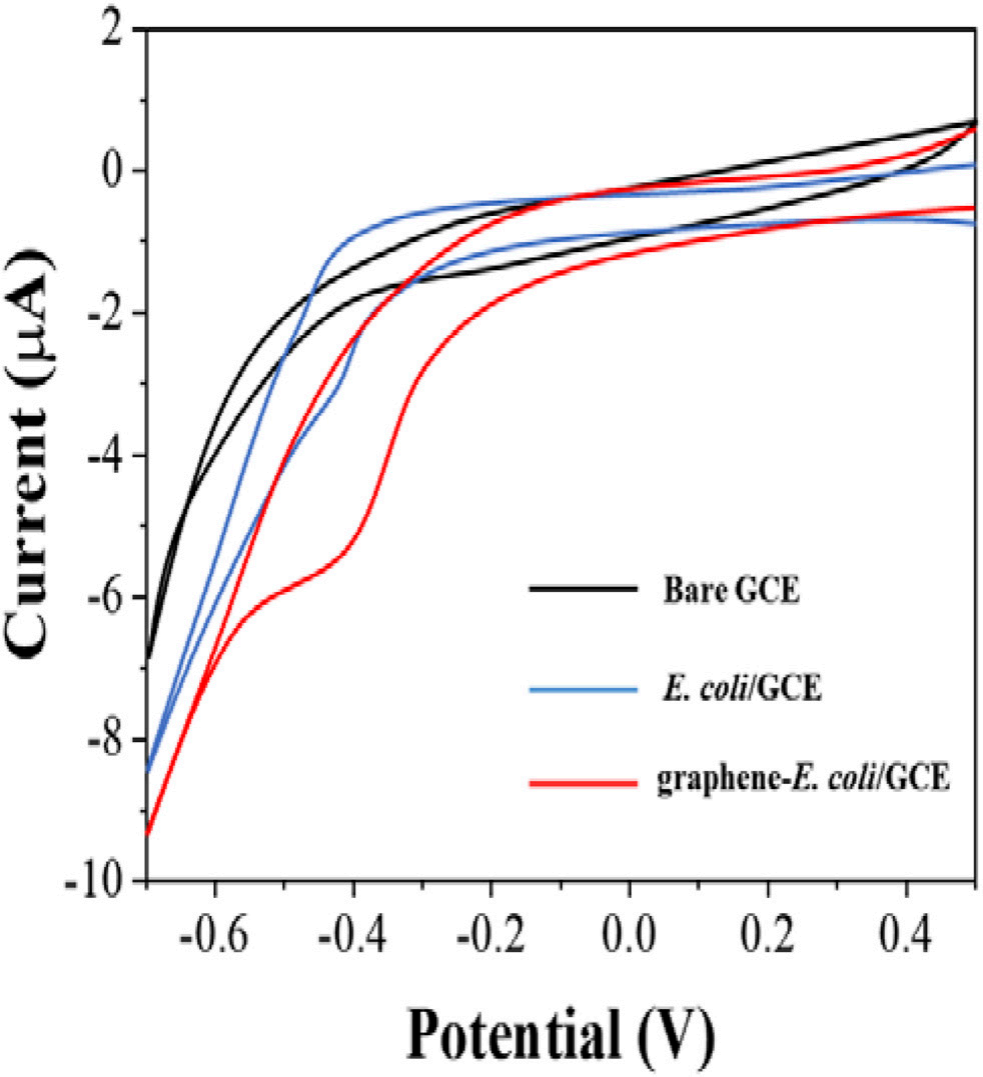
CVs of bare GCE, *E. coli*/GCE and graphene-*E. coli*/GCE in PBS (pH = 7).

Since bacterial cells have their own oxidoreductase system, which has been shown to be involved in electron transfer (Couto et al., 2018), we first investigated the direct electrochemical behavior of *E. coli*. First, we performed cyclic voltammetry (CV) tests only × 10^7^ CFU *E coli* with directly coated on the GCE surface (Figure 1). Comparing to bare GCE, we could see a clear reduction peak at around −0.4 V, which indicates that the electroactivity of bacterial cells undergoes surface electrode reaction. However, the reduction current of this reduction peak is not particularly pronounced and is only 3.2 μA. In contrast, the intensity of the reduction current of *E. coli* is significantly higher after co-mixing with graphene. There are two reasons for this increase. The first one is that the excellent electrical properties of graphene itself improve the electron transfer rate (Pourmadadi et al., 2019). The second is that the lamellar structure of graphene greatly enhances the electrochemically active area (Gupta et al., 2019). It enables more cells to participate in the electrochemical reaction after wrapping *E. coli*. Therefore, with the assistance of graphene, it became possible to evaluate the antibiotic resistance of *E. coli* cells from its electrochemical behavior.

**FIGURE 2:**
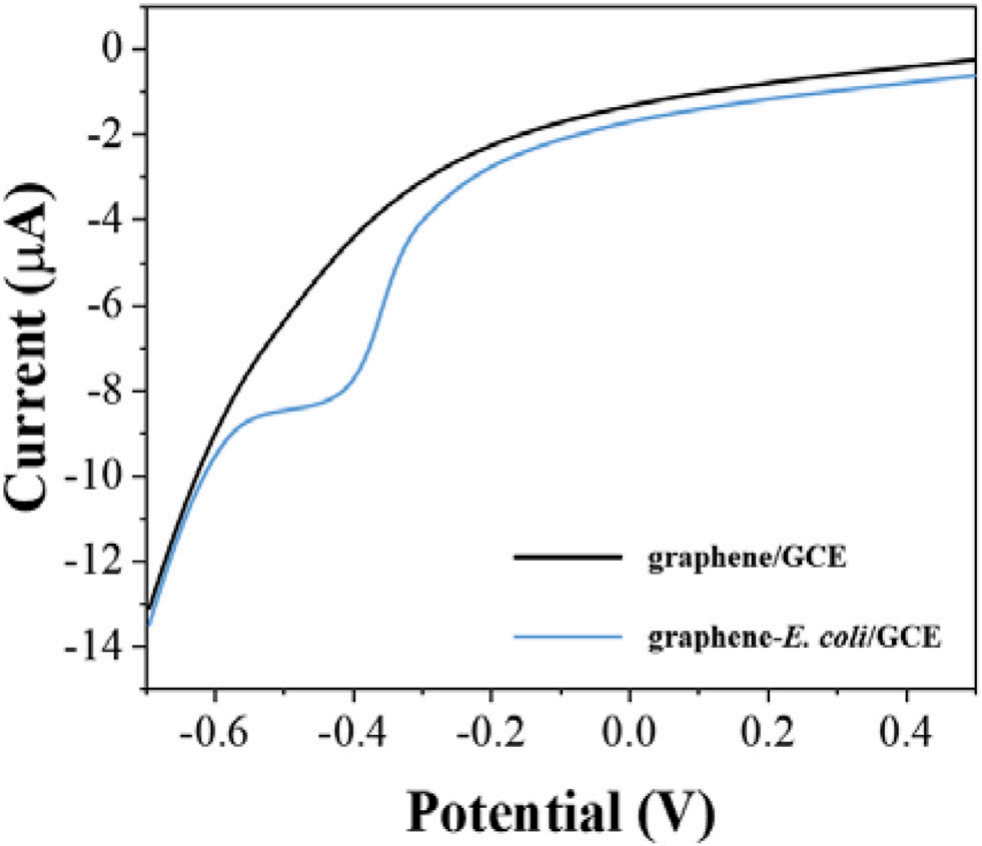
DPV curves of graphene/GCE and graphene-*E. coli*/GCE in PBS (pH = 7).

After determining the electrochemical behavior of *E. coli*, we used the electrochemical reduction peak as a probe for cell viability evaluation. To make the detection more sensitive, we further investigated the electrodes with differential pulse voltammetry (DPV). Figure 2 shows the DPV curves of graphene/GCE and graphene-*E. coli*/GCE. It can be seen that graphene/GCE shows only a flat curve, but the curve of graphene-*E. coli*/GCE has a clear reduction peak. At the same time, the reduction peak on DPV has some shift against CV, which is due to the amplitude added by DPV (Vilas-Boas et al., 2019). We can see that the DPV test has a better signal-to-noise ratio than the CV. This reduction reaction is catalyzed by some macromolecules in *E. coli* cells. The substances involved may be cell surface c-type cytochromes and bacterial outer membrane reductases, dehydrogenases and flavoproteins (Vinod et al., 2002).

**FIGURE 3:**
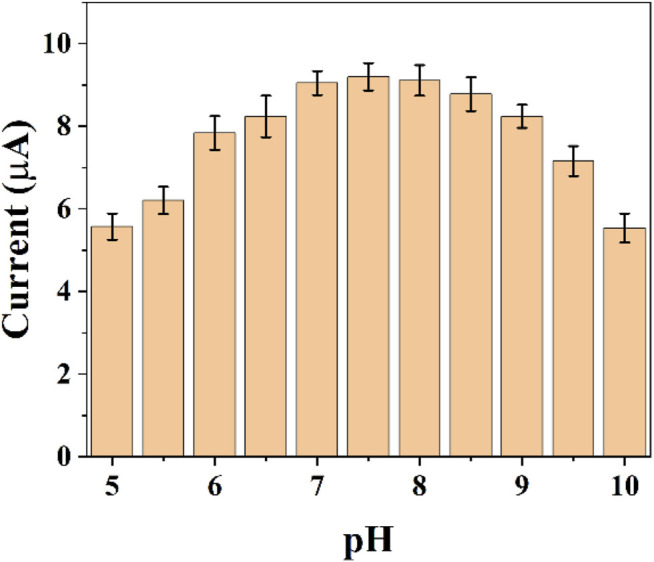
Reduction peak current of graphene-*E. coli*/GCE at different pH conditions (*n* = 3).

The pH of the buffer solution can significant effect on electrochemistry. Electrochemically active substances have different electrochemical behaviors at different pH conditions. In the same time, the activity of *E. coli* is different in different pH environments. Therefore, it is necessary for us to optimize the pH conditions. Figure 3 shows the difference of reduction currents between pH 5–10. It can be seen that the intensity of the currents gradually increases as the acidic conditions move toward the neutral conditions. The current peaks reached the maximum at 7.5. As the pH environment gradually becomes alkaline, the current value of the reduction peak starts to decrease. We finally chose the optimal pH environment as 7.5.

**FIGURE 4:**
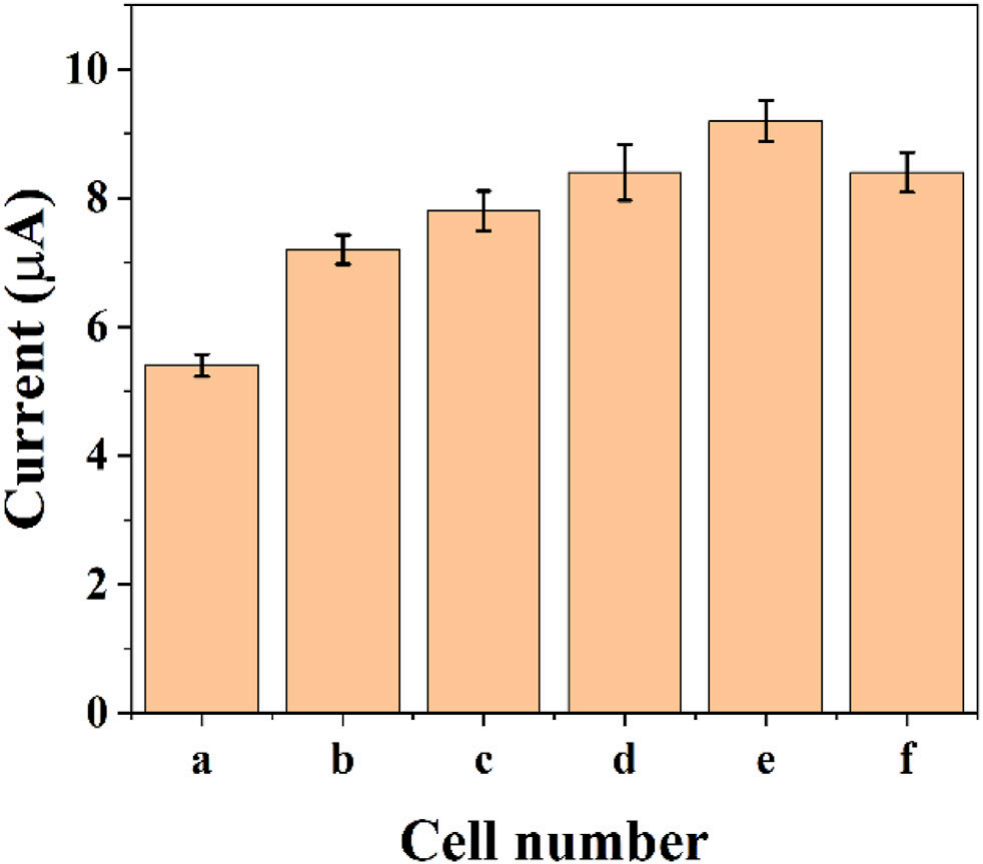
Reduction peak current of graphene-*E. coli*/GCE at PBS with 1 × 10^5^ CFU **(A)**, 5 × 10^5^ CFU **(B)**, 1 × 10^6^ CFU **(C)**, 5 × 10^6^ CFU **(D)**, 1 × 10^7^ CFU **(E)**, and 5 × 10^7^ CFU **(F)** (*n* = 3).

The reduction current of DPV will also increase due to the increase in the number of cells. Figure 4 shows the assay with graphene-*E. coli*/GCE for 1 × 10^5^ CFU, 5 × 10^5^ CFU, 1 × 10^6^ CFU, 5 × 10^6^ CFU, 1 × 10^7^ CFU, and 5 × 10^7^ CFU. It can be seen that the reduction current increases as the number of cells increases. This may be due to the fact that more cells are involved in the electrochemical reaction. However, too many cells also lead to a decrease in the current, which is due to the fact that *E. coli* itself does not have a good conductivity. Too many cells form a thicker film, which hinders the transfer of electrons. These results are in accordance with works published recent years regards to the electrochemistry of *E. coli* cells (Setterington and Alocilja, 2011; Dos Santos et al., 2013). To reveal the maximum variability, we chose 1 × 10^7^ CFU as the optimal condition.

**FIGURE 5:**
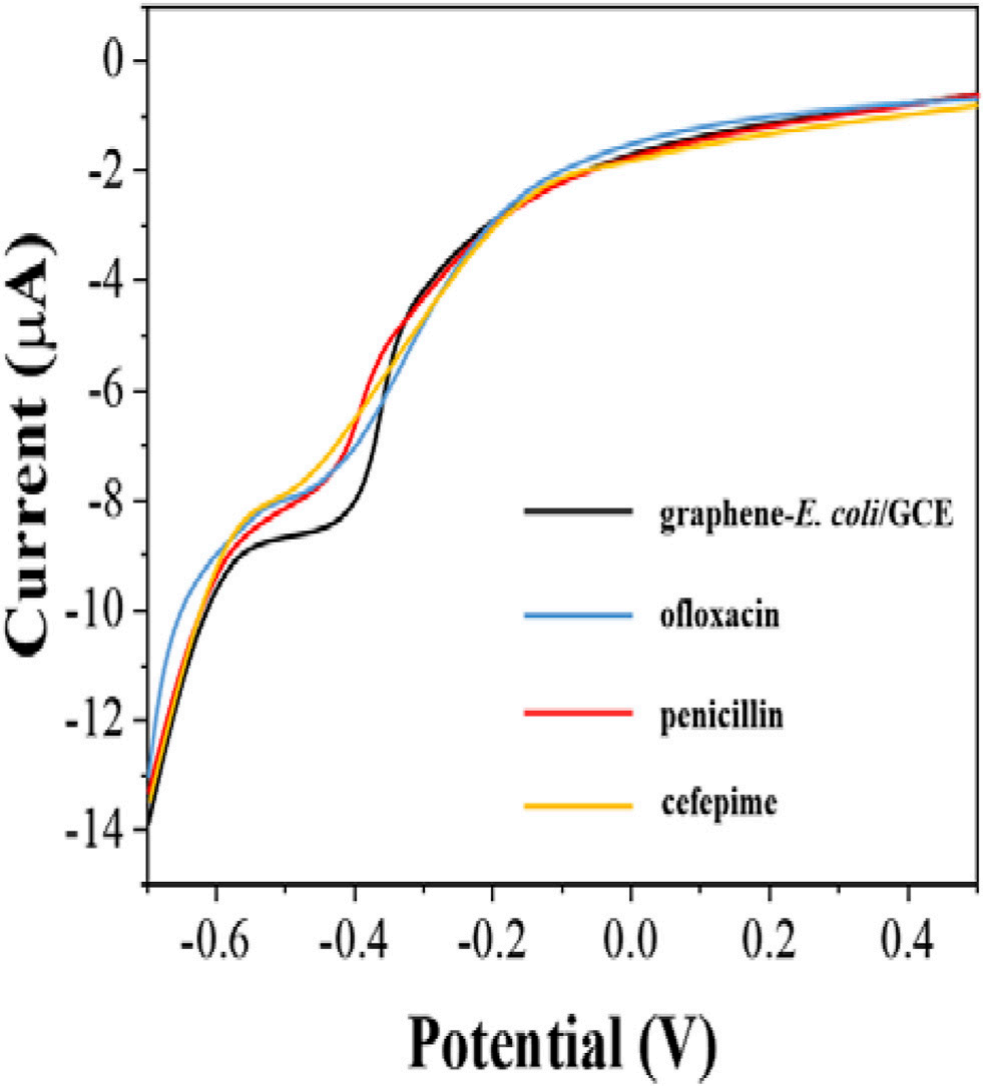
DPV curves of graphene-*E. coli*/GCE before and after addition of ofloxacin, penicillin and cefepime at PBS.

Since antibiotics can kill *E. coli*. The inactive *E. coli* is unable to perform effective electrochemical catalytic reaction. Therefore, the difference in reduction current can be used to detect the number of surviving *E. coli* on the electrode surface. However, *E. coli* possessing antibiotic resistance can survive in the presence of antibiotics and therefore the behavior of electrochemical reduction will receive only a small effect. In this work, we tested the susceptibility of *E. coli* to ofloxacin, penicillin and cefepime. Figure 5 shows the electrochemical behavior of graphene-*E. coli*/GCE 1 h after the addition of ofloxacin, penicillin and cefepime. It can be seen that there is a corresponding decrease in the reduction current in each curve compared to the electrochemical behavior without the addition of antibiotics. It represents a decrease in the number of cells able to participate in the electrochemically catalyzed reduction due to the destruction of *E. coli* by antibiotics and therefore a decrease in the current.

**FIGURE 6:**
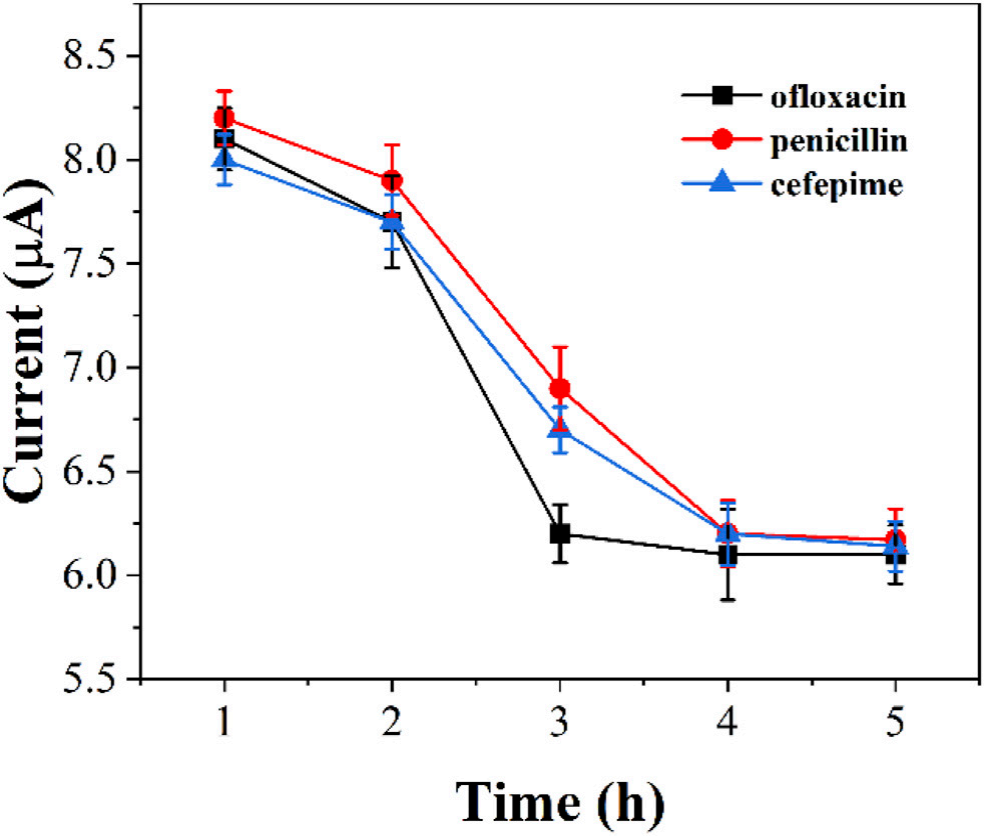
Reduction peak currents of graphene-*E. coli*/GCE at PBS after addition of ofloxacin, penicillin and cefepime (*n* = 3).

We monitored the bacterial inhibition of the three antibiotics. Figure 6 shows the electrochemical reduction currents at different times after the addition of antibiotics to graphene-*E. coli*/GCE. It can be seen that the electrochemical reduction current increases with time, indicating that the antibiotic continues to have an effect on *E. coli.* Ofloxacin after about 3 h The reduction current has no longer changes after 3 h after the addition of ofloxacin. The same was true for penicillin and cefepime, which took about 4 h. We can observe a gradual loss of activity of *E. coli* during this process. However, if *E. coli* has antibiotic resistance, it can maintain the original intensity of the reduction current. Therefore, this technique could potentially be used to identify drug-resistant strains of *E. coli*.

## Conclusion

In this work, we coated *E. coli* cells with a graphene dispersion, which was then immobilized on the electrode surface. This approach allows the evaluation of the activity of *E. coli* on the electrode surface. The electrocatalytic reduction current is the indicator in this evaluation. The current is proportional to the activity of the cells on the surface of the electrode according to the electrode. The antibiotic has an effect on the cells that result in the decreasing of the electrocatalytic reduction signal. Therefore, this strategy can be used to evaluate the resistance of cells to antibiotics. After optimization of the parameters, we successfully evaluated the resistance of *E. coli* to ofloxacin, penicillin and cefepime.

## Data Availability

The original contributions presented in the study are included in the article/Supplementary Material, further inquiries can be directed to the corresponding author.

## References

[B1] Abdullah A. Asghar A. Butt M. S. Shahid M. Huang Q. (2017). Evaluating the Antimicrobial Potential of green Cardamom Essential Oil Focusing on Quorum Sensing Inhibition of Chromobacterium Violaceum. J. Food Sci. Technol. 54, 2306–2315. 10.1007/s13197-017-2668-7 28740287 PMC5502022

[B2] Alsaiari N. S. Katubi K. M. M. Alzahrani F. M. Siddeeg S. M. Tahoon M. A. (2021). The Application of Nanomaterials for the Electrochemical Detection of Antibiotics: A Review. Micromachines 12, 308. 10.3390/mi12030308 33804280 PMC8000799

[B3] Baghayeri M. (2017). Pt Nanoparticles/reduced Graphene Oxide Nanosheets as a Sensing Platform: Application to Determination of Droxidopa in Presence of Phenobarbital. Sensors Actuators B: Chem. 240, 255–263. 10.1016/j.snb.2016.08.161

[B4] Bagheri S. S. Peighambari S. M. Soltani M. Malekan M. (2019). RAPD-PCR and Drug Resistance Pattern of *Staphylococcus aureus* Isolates Recovered from Companion and Wild Birds. Iranj. Vet. Med. 13, 356–364. 10.22059/ijvm.2019.282080.1004991

[B5] Bengtson H. N. Homolka S. Niemann S. Reis A. J. da Silva P. E. Gerasimova Y. V. (2017). Multiplex Detection of Extensively Drug Resistant Tuberculosis Using Binary Deoxyribozyme Sensors. Biosens. Bioelectron. 94, 176–183. 10.1016/j.bios.2017.02.051 28284077 PMC5407088

[B6] Chotinantakul K. Suginta W. Schulte A. (2014). Advanced Amperometric Respiration Assay for Antimicrobial Susceptibility Testing. Anal. Chem. 86, 10315–10322. 10.1021/ac502554s 25222107

[B7] Couto R. A. S. Chen L. Kuss S. Compton R. G. (2018). Detection of *Escherichia coli* Bacteria by Impact Electrochemistry. Analyst 143, 4840–4843. 10.1039/c8an01675e 30238951

[B8] Dos Santos M. B. Agusil J. Prieto-Simón B. Sporer C. Teixeira V. Samitier J. (2013). Highly Sensitive Detection of Pathogen *Escherichia coli* O157: H7 by Electrochemical Impedance Spectroscopy. Biosens. Bioelectron. 45, 174–180. 10.1016/j.bios.2013.01.009 23500360

[B9] Ertl P. Unterladstaetter B. Bayer K. Mikkelsen S. R. (2000). Ferricyanide Reduction byEscherichiacoli: Kinetics, Mechanism, and Application to the Optimization of Recombinant Fermentations. Anal. Chem. 72, 4949–4956. 10.1021/ac000358d 11055714

[B10] Ertl P. Wagner M. Corton E. Mikkelsen S. R. (2003). Rapid Identification of Viable *Escherichia coli* Subspecies with an Electrochemical Screen-Printed Biosensor Array. Biosens. Bioelectron. 18, 907–916. 10.1016/s0956-5663(02)00206-3 12713914

[B11] Farooq U. Ullah M. W. Yang Q. Aziz A. Xu J. Zhou L. (2020). High-density Phage Particles Immobilization in Surface-Modified Bacterial Cellulose for Ultra-sensitive and Selective Electrochemical Detection of *Staphylococcus aureus* . Biosens. Bioelectron. 157, 112163. 10.1016/j.bios.2020.112163 32250935

[B12] Fu Y. Zhou X. Duan X. Liu C. Huang J. Zhang T. (2020). A LAMP-Based Ratiometric Electrochemical Sensing for Ultrasensitive Detection of Group B Streptococci with Improved Stability and Accuracy. Sensors Actuators B: Chem. 321, 128502. 10.1016/j.snb.2020.128502

[B13] Gorlenko C. L. Kiselev H. Y. Budanova E. V. Zamyatnin A. A. Ikryannikova L. N. (2020). Plant Secondary Metabolites in the Battle of Drugs and Drug-Resistant Bacteria: New Heroes or Worse Clones of Antibiotics? Antibiotics 9, 170. 10.3390/antibiotics9040170 PMC7235868 32290036

[B14] Gupta A. Bhardwaj S. K. Sharma A. L. Deep A. (2019). A Graphene Electrode Functionalized with Aminoterephthalic Acid for Impedimetric Immunosensing of *Escherichia coli* . Microchim. Acta 186, 1–7. 10.1007/s00604-019-3952-1 31741076

[B15] Hu C. Kalsi S. Zeimpekis I. Sun K. Ashburn P. Turner C. (2017). Ultra-fast Electronic Detection of Antimicrobial Resistance Genes Using Isothermal Amplification and Thin Film Transistor Sensors. Biosens. Bioelectron. 96, 281–287. 10.1016/j.bios.2017.05.016 28505562

[B16] Hu K. Huang X. Jiang Y. Fang W. Yang X. (2010). Monoclonal Antibody Based Enzyme-Linked Immunosorbent Assay for the Specific Detection of Ciprofloxacin and Enrofloxacin Residues in Fishery Products. Aquaculture 310, 8–12. 10.1016/j.aquaculture.2010.08.008

[B17] Karimi-Maleh H. Alizadeh M. Orooji Y. Karimi F. Baghayeri M. Rouhi J. (2021a). Guanine-Based DNA Biosensor Amplified with Pt/SWCNTs Nanocomposite as Analytical Tool for Nanomolar Determination of Daunorubicin as an Anticancer Drug: A Docking/Experimental Investigation. Ind. Eng. Chem. Res. 60, 816–823. 10.1021/acs.iecr.0c04698

[B18] Karimi-Maleh H. Karimi F. Malekmohammadi S. Zakariae N. Esmaeili R. Rostamnia S. (2020). An Amplified Voltammetric Sensor Based on Platinum Nanoparticle/polyoxometalate/two-Dimensional Hexagonal boron Nitride Nanosheets Composite and Ionic Liquid for Determination of N-Hydroxysuccinimide in Water Samples. J. Mol. Liquids 310, 113185. 10.1016/j.molliq.2020.113185

[B19] Karimi-Maleh H. Orooji Y. Karimi F. Alizadeh M. Baghayeri M. Rouhi J. (2021b). A Critical Review on the Use of Potentiometric Based Biosensors for Biomarkers Detection. Biosens. Bioelectron. 184, 113252. 10.1016/j.bios.2021.113252 33895688

[B20] Karimi-Maleh H. Yola M. L. Atar N. Orooji Y. Karimi F. Senthil Kumar P. (2021c). A Novel Detection Method for Organophosphorus Insecticide Fenamiphos: Molecularly Imprinted Electrochemical Sensor Based on Core-Shell Co3O4@MOF-74 Nanocomposite. J. Colloid Interf. Sci. 592, 174–185. 10.1016/j.jcis.2021.02.066 33662823

[B21] Khan M. Z. H. Hasan M. R. Hossain S. I. Ahommed M. S. Daizy M. (2020). Ultrasensitive Detection of Pathogenic Viruses with Electrochemical Biosensor: State of the Art, 166. Biosens. Bioelectron. 112431. 10.1016/j.bios.2020.112431 32862842 PMC7363606

[B22] Mishra M. Arukha A. P. Patel A. K. Behera N. Mohanta T. K. Yadav D. (2018). Multi-drug Resistant Coliform: Water Sanitary Standards and Health Hazards. Front. Pharmacol. 9, 311. 10.3389/fphar.2018.00311 29946253 PMC6005870

[B23] Mohanraj J. Durgalakshmi D. Rakkesh R. A. Balakumar S. Rajendran S. Karimi-Maleh H. (2020). Facile Synthesis of Paper Based Graphene Electrodes for point of Care Devices: a Double Stranded DNA (dsDNA) Biosensor. J. Colloid Interf. Sci. 566, 463–472. 10.1016/j.jcis.2020.01.089 32032811

[B24] Mulat M. Pandita A. Khan F. (2019). Medicinal Plant Compounds for Combating the Multi-Drug Resistant Pathogenic Bacteria: a Review. Cpb 20, 183–196. 10.2174/1872210513666190308133429 30854956

[B25] Osman K. M. da Silva Pires Á. Franco O. L. Saad A. Hamed M. Naim H. (2021). Nile tilapia (*Oreochromis niloticus*) as an Aquatic Vector for Pseudomonas Species of Medical Importance: Antibiotic Resistance Association with Biofilm Formation, Quorum Sensing and Virulence. Aquaculture 532, 736068. 10.1016/j.aquaculture.2020.736068

[B26] Phung T. T. B. Chu S. V. Vu S. T. Pham H. T. Nguyen H. M. Nguyen H. D. (2020). COLD-PCR Method for Early Detection of Antiviral Drug-Resistance Mutations in Treatment-Naive Children with Chronic Hepatitis B. Diagnostics 10, 491. 10.3390/diagnostics10070491 PMC7400161 32708399

[B27] Pourmadadi M. Shayeh J. S. Omidi M. Yazdian F. Alebouyeh M. Tayebi L. (2019). A Glassy Carbon Electrode Modified with Reduced Graphene Oxide and Gold Nanoparticles for Electrochemical Aptasensing of Lipopolysaccharides from *Escherichia coli* Bacteria. Microchim. Acta 186, 1–8. 10.1007/s00604-019-3957-9 31732807

[B28] Sedki M. Hassan R. Y. A. Hefnawy A. El-Sherbiny I. M. (2017). Sensing of Bacterial Cell Viability Using Nanostructured Bioelectrochemical System: rGO-Hyperbranched Chitosan Nanocomposite as a Novel Microbial Sensor Platform. Sensors Actuators B: Chem. 252, 191–200. 10.1016/j.snb.2017.05.163

[B29] Setterington E. B. Alocilja E. C. (2011). Rapid Electrochemical Detection of Polyaniline-Labeled *Escherichia coli* O157:H7. Biosens. Bioelectron. 26, 2208–2214. 10.1016/j.bios.2010.09.036 20956078

[B30] Sharaha U. Rodriguez-Diaz E. Riesenberg K. Bigio I. J. Huleihel M. Salman A. (2017). Using Infrared Spectroscopy and Multivariate Analysis to Detect Antibiotics' ResistantEscherichia coliBacteria. Anal. Chem. 89, 8782–8790. 10.1021/acs.analchem.7b01025 28731324

[B31] Simioni N. B. Silva T. A. Oliveira G. G. Fatibello-Filho O. (2017). A Nanodiamond-Based Electrochemical Sensor for the Determination of Pyrazinamide Antibiotic. Sensors Actuators B: Chem. 250, 315–323. 10.1016/j.snb.2017.04.175

[B32] Sun Y. Zhao C. Niu J. Ren J. Qu X. (2020). Colorimetric Band-Aids for point-of-care Sensing and Treating Bacterial Infection. ACS Cent. Sci. 6, 207–212. 10.1021/acscentsci.9b01104 32123738 PMC7047266

[B33] Vilas-Boas Â. Valderrama P. Fontes N. Geraldo D. Bento F. (2019). Evaluation of Total Polyphenol Content of Wines by Means of Voltammetric Techniques: Cyclic Voltammetry vs Differential Pulse Voltammetry. Food Chem. 276, 719–725. 10.1016/j.foodchem.2018.10.078 30409654

[B34] Vinod M. P. Bellur P. Becker D. F. (2002). Electrochemical and Functional Characterization of the Proline Dehydrogenase Domain of the PutA Flavoprotein fromEscherichia Coli†. Biochemistry 41, 6525–6532. 10.1021/bi025706f 12009917

[B35] Wang M. Hu M. Liu J. Guo C. Peng D. Jia Q. (2019). Covalent Organic Framework-Based Electrochemical Aptasensors for the Ultrasensitive Detection of Antibiotics. Biosens. Bioelectron. 132, 8–16. 10.1016/j.bios.2019.02.040 30851495

[B36] Wang Y. Dong W. Odah K. A. Kong L. Ma H. (2019). Transcriptome Analysis Reveals AI-2 Relevant Genes of Multi-Drug Resistant *Klebsiella pneumoniae* in Response to Eugenol at Sub-MIC. Front. Microbiol. 10, 1159. 10.3389/fmicb.2019.01159 31191486 PMC6547871

[B37] Waseem H. Jameel S. Ali J. Saleem Ur Rehman H. Tauseef I. Farooq U. (2019). Contributions and Challenges of High Throughput qPCR for Determining Antimicrobial Resistance in the Environment: a Critical Review. Molecules 24, 163. 10.3390/molecules24010163 PMC6337382 30609875

[B38] Wu W. Wu M. Zhou J. Xu Y. Li Z. Yao Y. (2020). Development of Electrochemical Sensor for Fast Liquor Authentication. Sens. Mater. 32, 2941–2948. 10.18494/sam.2020.2972

[B39] Xu Y. Lu Y. Zhang P. Wang Y. Zheng Y. Fu L. (2020). Infrageneric Phylogenetics Investigation of Chimonanthus Based on Electroactive Compound Profiles. Bioelectrochemistry 133, 107455. 10.1016/j.bioelechem.2020.107455 31978859

[B40] Zhang L. Liang W. Ran Q. Liu F. Chen D. Xiong Y. (2020). Ultrasensitive Detection of NDM-1 Resistant Bacteria Based on Signal Amplification with sandwich-type LNA Electrochemical Biochips. Sensors Actuators B: Chem. 306, 127556. 10.1016/j.snb.2019.127556

[B41] Zhang M. Pan B. Wang Y. Du X. Fu L. Zheng Y. (2020). Recording the Electrochemical Profile of Pueraria Leaves for Polyphyly Analysis. ChemistrySelect 5, 5035–5040. 10.1002/slct.202001100

[B42] Zhang X. Yang R. Li Z. Zhang M. Wang Q. Xu Y. (2020). Electroanalytical Study of Infrageneric Relationship of Lagerstroemia Using Glassy Carbon Electrode Recorded Voltammograms. Rmiq 19, 281–291. 10.24275/rmiq/bio1750

[B43] Zhao L. Liu Y. Zhang Z. Wei J. Xie S. Li X. (2020). Fibrous Testing Papers for Fluorescence Trace Sensing and Photodynamic Destruction of Antibiotic-Resistant Bacteria. J. Mater. Chem. B 8, 2709–2718. 10.1039/d0tb00002g 32149315

[B44] Zhi-bin L. Min W. Xiao-cui W. Min H. He-ping X. Qing Z. (2021). The Value of PCR-Reverse Dot Blot Hybridization in Detecting the Drug Resistance of *Mycobacterium tuberculosis* in Sputum Specimens of Retreatment Smear-Positive Pulmonary Tuberculosis Patients. Chin. J. Antituberc. 43, 47. 10.17343/sdutfd.534941

[B45] Zhou T. Han H. Liu P. Xiong J. Tian F. Li X. (2017). Microbial Fuels Cell-Based Biosensor for Toxicity Detection: A Review. Sensors 17, 2230. 10.3390/s17102230 PMC5677232 28956857

[B46] Zhuang J. Wang S. Tan Y. Xiao R. Chen J. Wang X. (2019). Degradation of Sulfadimethoxine by Permanganate in Aquatic Environment: Influence Factors, Intermediate Products and Theoretical Study. Sci. Total Environ. 671, 705–713. 10.1016/j.scitotenv.2019.03.277 30939323

